# The vasopressin Avprlb receptor: Molecular and pharmacological studies

**DOI:** 10.3109/10253890.2010.512376

**Published:** 2010-09-10

**Authors:** JA Roper, A-M O'Carroll, WS Young, SJ Lolait

**Affiliations:** 1 Henry Wellcome LINE, University of Bristol, Dorothy Hodgkin Building, Whitson Street, Bristol BS1 3NY, UK; 2 Section on Neural Gene Expression, Department of Health and Human Services, National Institute of Mental Health, National Institutes of Health, Bethesda, MA 20892-4483, USA

**Keywords:** Aggression, hypothalamic–pituitary–adrenal axis, stress, Avprlb receptor, Avprlb antagonist, vasopressin

## Abstract

The distribution, pharmacology and function of the arginine vasopressin (Avp) lb receptor subtype (Avprlb) has proved more challenging to investigate compared to other members of the Avp receptor family. Avp is increasingly recognised as an important modulator of the hypothalamic–pituitary–adrenal (HPA) axis, an action mediated by the Avprlb present on anterior pituitary corticotrophs. The Avprlb is also expressed in some peripheral tissues including pancreas and adrenal, and in the hippocampus (HIP), paraventricular nucleus and olfactory bulb of the rodent brain where its function is unknown. The central distribution of Avprlbs is far more restricted than that of the Avprla, the main Avp receptor subtype found in the brain. Whether Avprlb expression in rodent tissues is dependent on differences in the length of microsatellite dinucleotide repeats present in the 5′ promoter region of the Avprlb gene remains to be determined. One difficulty of functional studies on the Avprlb, especially its involvement in the HPA axis response to stress, which prompted the generation of Avprlb knockout (KO) mouse models, was the shortage of commercially available Avprlb ligands, particularly antagonists. Research on mice lacking functional Avprlbs has highlighted behavioural deficits in social memory and aggression. The Avprlb KO also appears to be an excellent model to study the contribution of the Avprlb in the HPA axis response to acute and perhaps some chronic (repeated) stressors where corticotrophin-releasing hormone and other genes involved in the HPA axis response to stress do not appear to compensate for the loss of the Avprlb.

## Introduction

Arginine vasopressin (Avp) is a cyclic nonapeptide that exerts diverse biological effects through a number of receptors (R), three of which have been cloned to date in mammals; the vasopressin Avprla, Avprlb and Avpr2—Avp also binds to the structurally related oxytocin (Oxt) receptor (Oxtr) with high affinity. Each member of the Avp receptor family has a discrete peripheral distribution and function; however, data for the central distribution of these receptors are still incomplete. The Avp receptor family are G protein-coupled receptors: the Avprla and Avprlb subtypes are both coupled to G_q/11_ and signal via phospholipase C (Jard et al. 1987; Thibonnier et al. 2001). The Avpr2 receptor subtype is coupled to G_s_ which, when activated, elevates cAMP levels by recruiting adenylate cyclase. It should be noted that many G protein-coupled receptors (GPCRs) couple to multiple signal transduction pathways, including the Avprlb (Thibonnier et al. 2001)—this may be related to the number of receptors and signal transduction components expressed in a given cell. The Oxtr predominantly signals via G_q/11_ but is a promiscuous receptor in that it may signal through a variety of different α subunits (Reversi et al. 2005; Chini and Manning 2007). The tissue distribution and physiological function of each receptor demonstrates Avp's primary function in fluid balance and homeostasis. The Avprla is predominantly found in vascular smooth muscle where it is involved in maintaining blood pressure via its classical pressor action (Koshimizu et al. 2006). The renal Avpr2 is responsible for water resorption in kidney collecting ducts by promoting the translocation of aquaporin-2 channels to the plasma membrane (Knepper 1997). The Avprlb is primarily located in the anterior lobe corticotrophs of the pituitary gland (Jard et al. 1986; Lolait et al. 1995). Avp in hypophysial portal blood acts on pituitary Avpr1bs to release adrenocorticotrophic hormone (ACTH) as part of the neuroendocrine response to stress (Antoni 1993). The contraction of uterine smooth muscle during parturition and milk let down during lactation are actions mediated by Oxt and the Oxtr. The distribution and function of Avp receptors is not limited to those sites mentioned above and all receptors except the Avpr2 are expressed in the brain. The central role each of these receptors plays has not yet been fully elucidated but the growing amount of data from pharmacological and knockout (KO) studies suggests some functional overlap (e.g. in modulating some social behaviour).

The availability of several specific ligands for these receptors has considerably aided in the characterisation of the Avpr1a, Avpr2 and Oxtr but, until recently, research on the Avpr1b has been hampered by a lack of Avpr1b-specific ligands. Avpr1b research has focussed primarily on the detection of receptor transcript levels (indirectly inferring receptor protein levels), genetic KO studies and experiments with the naturally Avp-deficient Brattleboro (di/di) rats. This review details recent pharmacological and KO data on the role of the Avpr1b in brain, pituitary and peripheral tissues with particular emphasis on its function in the hypothalamic–pituitary–adrenal (HPA) axis response to stress.

## V1B receptor distribution

The Avpr 1 a and the Oxtr have been well characterised in the rodent brain and are thought to be the likely substrates for central actions of Avp and/or Oxt (de Wied et al. 1993; Burbach et al. 1995; Barberis and Tribollet 1996). More recent data from KO animals suggest specific behavioural deficits in social memory and aggression are directly due to the absence of central Avpr1bs (Wersinger et al. 2002). The search for central Avpr1bs has proved more elusive than that of the Avpr 1as and Oxtrs as the shortage of specific ligands has prevented binding studies to visualise the Avpr1b protein. Nevertheless, a number of studies utilising immunohistochemistry and *in situ* hybridisation histochemistry (ISHH) have shown that the Avpr1b is expressed centrally, while reverse transcription-polymerase chain reaction (RT-PCR) and functional studies have demonstrated Avpr1bs in a number of peripheral tissues (e.g. Lolait et al. 1995; Vaccari et al. 1998; Hernando et al. 2001; O'Carroll et al. 2008).

Although the highest concentration of Avpr1bs is found within anterior pituitary corticotrophs (Jard et al. 1986; Antoni 1993; Lolait et al. 1995), several studies suggest a wide central (Barberis and Tribollet 1996; Vaccari et al. 1998; Hernando et al. 2001) and peripheral (Lolait et al. 1995; Saito et al. 1995; Ventura et al. 1999; Oshikawa et al. 2004) distribution in rodents. Analysis of various brain regions and peripheral tissues suggests that Avpr1b transcript levels may be too low to be reliably detected by Northern blot analysis and often depends on RT-PCR to detect possible Avpr1b expression (Lolait et al. 1995). A recent distribution study using ISHH with probes directed to 5′ or 3′ untranslated regions of the Avpr1b mRNA details a more restricted pattern of Avpr1b mRNA in mouse brain than previously reported (Young et al. 2006). The riboprobes used by Young and co-workers had low sequence identity to other Avp receptors to minimise cross-reactivity with related mRNA sequences. This study shows Avpr1b mRNA to be most prominent in the CA2 pyramidal neurons of the mouse and human HIP while receptor transcripts are also found in the paraventricular nucleus (PVN) and amygdala, albeit at a lower level. All of these studies infer receptor expression by determining mRNA transcript levels rather than receptor protein levels. However, in the absence of specific radiolabelled ligands of high-specific activity (which may not provide detailed anatomical resolution), mRNA expression coupled with immunohis-tochemical techniques can accurately reflect receptor protein distribution and quantity.

The peripheral distribution of the Avpr1b is much more restricted than that of the Avpr1a, which is ubiquitously expressed (Oshikawa et al. 2004; Fujiwara et al. 2007a). Avpr1b mRNA has been detected by RT-PCRin the rodent pancreas (Saito et al. 1995; Ventura et al. 1999; Oshikawa et al. 2004), adrenal gland (Grazzini et al. 1996; Ventura et al. 1999; Oshikawa et al. 2004), spleen (Lolait et al. 1995; Oshikawa et al. 2004), kidney (Lolait et al. 1995; Saito et al. 1995), heart (Lolait et al. 1995; Saito et al. 1995), liver (Saito et al. 1995) and lung (Lolait et al. 1995; Saito et al. 1995). Additionally, the thymus (Lolait et al. 1995), colon (Ventura et al. 1999), small intestine, bladder (Saito et al. 1995), breast and uterus (Lolait et al. 1995) and white adipose tissue (Fujiwara et al. 2007a) reportedly contain Avpr1b mRNA, however, these have not been ratified by all studies (e.g. only one out of five studies observe Avpr1b mRNA in rodent liver). In any event, the functional significance of varying amounts of low levels of Avpr 1b mRNA detected by RT-PCR in whole brain or peripheral tissue samples is unknown. Disparities between laboratories may reflect methodological (e.g. detection of amplified PCR products) and/or strain differences. The mRNA-expressing tissues where there appears to be strong functional correlations are the pancreas and adrenal gland.

In the pancreas, Avp has been shown to act on Avpr1bs present in islets to stimulate the secretion of insulin from β cells where it may act synergistically with corticotrophin-releasing hormone (Crh) (Oshikawa et al. 2004; O'Carroll et al. 2008). While Avp acts on the Avpr1b to decrease blood sugar levels through insulin release, it can also act in opposition to this by stimulating glucagon release (Yibchok-anun and Hsu 1998) and promoting hepatic glycogenolysis (Kirk et al. 1979). Importantly, Avp-mediated glycogenolysis acts via the Avpr1a subtype present in hepatic tissue (Morel et al. 1992) suggesting bifunctional but opposite roles of Avp in glucose homeostasis by employing two different receptor subtypes. Exactly which role Avp plays in regulating glucose balance at the pancreatic level is dependent on the local glucose concentration present in this tissue (Abu-Basha et al. 2002). One study on a glucagon-secreting hamster α-pancreatic cell line suggests that Avp-induced glucagon secretion is mediated via the Avpr1b since the Avpr1b antagonist SSR149415 potently antagonises Avp's effects in these cells (Folny et al. 2003). The specificity of SSR149415 has, however, been questioned with evidence of activity in Chinese hamster ovary cell lines expressing recombinant human Oxtrs (Griffante et al. 2005; Hodgson et al. 2007). This may be an important consideration, as Oxtrs in addition to Avpr1bs are apparently present in pancreatic islets (Oshikawa et al. 2004) and have been shown to cause insulin/glucagon release (Jeng et al. 1996; Yibchok-anun et al. 1999). One laboratory that used agonists and antagonists to Avp/Oxt receptors (but notably none selective for Avpr1bs) in α-cell lines (Yibchok-anun and Hsu 1998) and perfused rat pancreas (Yibchok-anun et al. 1999) produced contrasting results, the latter study suggesting glucagon secretion in response to Avp, and Oxt is mediated through the activation of Avpr1bs and Oxtrs, respectively, rather than any activity of Avp on Oxtrs. On the other hand, studies in Avpr1b KO mice suggest both Avp and Oxt can stimulate glucagon secretion through Oxtrs (Fujiwara et al. 2007b). Avp may play a role in the hypersecretion of glucagon from the pancreas of diabetics (Yibchok-anun et al. 2004), which is likely to involve the Avpr1b. Pancreatic β cells isolated from mice lacking functional Avpr1bs unsurprisingly display a blunted insulin secretion (Oshikawa et al. 2004), reinforcing the role of Avpr1bs in this tissue. Interestingly, further studies with this Avpr1b KO line show an increased sensitivity of Avpr1b KO mice to the metabolic effects of insulin (Fujiwara et al. 2007a). Together with the discovery of Avpr1b mRNA by RT-PCR in white adipose tissue, Fujiwara and co-workers suggest that the disruption in insulin–adipocyte signalling may lead to altered metabolism of glucose in Avpr1b KO mice. Whether this is due to a lack of Avpr1b influence in the pancreas, white adipose tissue, or both, is unclear.

Grazzini and co-workers demonstrated the presence of Avpr1b mRNA by RT-PCR in the medulla but not in the cortex of the rat adrenal gland. The cortex expresses Avpr1a transcripts primarily in the zona glomerulosa (Guillon et al. 1995; Grazzini et al. 1996) where this receptor regulates steroid secretion *in vitro* (Grazzini et al. 1998). These studies also show that Avp precursor mRNA and Avp peptide are present in the adrenal medulla suggesting Avp can be released within the tissue, possibly acting in an autocrine/paracrine manner to regulate adrenal function. Stimulation of the Avpr1b in the rat adrenal medulla causes catecholamine secretion (Grazzini et al. 1998). The presence of Avpr1as in the adrenal cortex and Avpr1bs in the chromaffin cells of the medulla provides strong evidence of an independent modulatory role of each receptor in discrete regions of adrenal tissue. The presence, however, of both Avpr1s in the human (Grazzini et al. 1999) adrenal medulla indicates a possible co-expression of Avpr1 receptors in some species. This suggests a possible overlap of function distinct from the roles already noted and may reflect the action of Avp originating from different sources (e.g. pituitary vs. local tissue release (Gallo-Payet and Guillon 1998)), although the medullary cell type that expresses Avpr1as has yet to be identified. Notably, the plasma catecholamine response to forced swimming and social isolation stress is attenuated in Avpr1b KO mice (Itoh et al. 2006).

**Figure 1:**
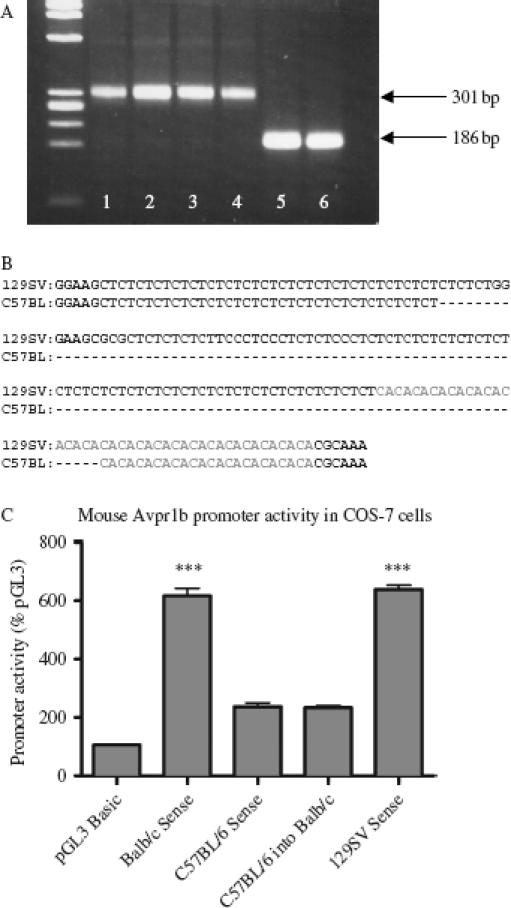
(A) PCR analysis of the microsatellite region in the 5′ promoter of the mouse Avprlb gene using genomic DNA from three different mouse strains (1,2, Balb/cOlaHsd; 3,4,129S2/SvHsd; 5,6, C57BI76JOlaHsd). The PCR products were generated using 100ng genomic DNA, 0.5 units of AmpliTaq polymerase (Applied Biosystems, Warrington, UK) and primers: upstream 5′ GC*G AGC TC*T TTC ACA CAT GCC TAG G 3′ incorporating SacI restriction site (underlined); downstream 5′ CA*G GAT CC*A CTG AGC ACC AAC TCA C 3′ incorporating a BamH1 restriction site (underlined) with cycling conditions of 95°C 1 min followed by 40 cycles of 94°C for 1 min, 62.5°C 1 min, 72°C 30 s followed by a final 72°C step for 10 min and a 4°C soak. Gel electrophoresis (2% agarose) of a 15-μl sample from the 50-μl reaction volume revealed a 301 bp product (bp1755–2055) with Balb/cOlaHsd and 129S2/SvHsd DNA templates and a ∼186 bp product with C57BL6J/OlaHsd strain DNA template. The PCR products were subcloned into pGEM4Z vector using Bam-H1 and Sac-1 restriction enzymes and sequenced. (B) Alignment of 129SV and C57BL strain microsatellite sequences between bases 1801 and 1988 of Genbank Acc#AF152533 (ending 824 bases upstream of the initiating ATG codon). Microsatellite CA and CT repeats are highlighted with dashes representing nucleotides that are absent in the C57BL mouse 5′ promoter region that gives rise to the shorter sequence. The longer form is present in 129/Svj, J1, SWR/J, AKR/J, FVB and CD1 (USA) 129S2/SvHsd and Balb/cOlaHsd (UK) strains, whereas the shorter form is in C57BL/6Ncr (USA), C57BL/6JOlaHsd (UK) and C57BI76J (USA) strains. (C) Fragments of 5′ Avprlb gene promoter region incorporating the microsatellite repeats were generated by PCR using 100ng genomic DNA, Herculase II Fusion DNA polymerase (Stratagene (Agilent Technologies), Stockport, UK), and primers corresponding to a ∼ 1.1 kb region of the mouse Avprlb gene (from bp1755–bp2823, Genbank accession number AF152533) using genomic DNA extracted from the Balb/c, C57BL/6 and 129 Sv mouse strains (Harlan, Bicester, UK). Amplified fragments from each strain were then subcloned into a pGL3 basic dual-luciferase reporter assay system (Promega, Southampton, UK) for expression in COS-7 cells. Values (mean ± SEM) are expressed as a percentage of control pGL3 basic activity. The promoter activity of vectors containing Balb/c and 129 Sv constructs is ∼ 3 × greater than that of C57BL/6 (*** = *P* < 0.001). When the Balb/c microsatellite region was replaced with the C57BL/6 microsatellite sequence in the Balb/c reporter construct, activity diminished to that of the C57BL/6 strain.

The central, pituitary and peripheral expression of the Avpr1b gene may be influenced by activity of elements in the upstream Avpr1b promoter region. *In vitro* studies using cells transiently transfected with a rat Avpr1b gene promoter sequence have identified regulatory GAGA repeats that influence Avpr1b gene transcription (Volpi et al. 2002). This provides a possible mechanism of physiological Avpr1b gene regulation that may enable different levels of Avpr1b expression in different tissues or species. When we compared the 5′ microsatellite region in the 5′ Avpr1b promoter sequence of different mouse strains, a major size difference in microsatellite length between the C57BL6J/OlaHsd strain and Balb/cOlaHsd and 129S2/SvHsd strains was observed (see, Figure 1A). Further analysis of the sequence details differences in the number of CTand C A repeats between strains (see, Figure 1B) that may confer changes in promoter activity. Basal promoter activity of a Balb/c 5′ fragment is threefold greater than that of the C57BL/6 strain confirming an increase in Avpr1b promoter activity with the “long” form of microsatellite (see, Figure 1B and C), in a reporter assay in COS-7 cells. The impact of microsatellite DNA sequences on receptor expression and behavioural phenotypes has been examined in studies on the effects of Avpr1a expression on social behaviour in voles. Affiliative behaviours such as pair bonding have been attributed to changes in Avpr1a expression patterns caused by microsatellite length variations in the 5′ Avpr1a regulatory region (Hammock and Young 2002; Hammock and Young 2005). Differences in Avpr1b protein expression that result from variances in gene promoter activity between mouse strains may be a contributing factor to varying susceptibility to stress. Several neurogenic, psychogenic and systemic stressors have been tested in different strains of mice to reveal a strain-dependant stress response (Anisman et al. 2001). Interestingly, C57BL/6ByJ mice display higher levels of plasma CORT as well as increases in stress-related behaviours compared to the Balb/cByJ strain, strengthening support for Avprlb's involvement in mouse stress susceptibility (Anisman et al. 2001). It is important to note, however, that similar 5′ microsatellite sequences are not present in the human Avprlb gene.

The relatively recent emergence of data from genetic KO studies and the development of promising pharmacological compounds have given the task of characterising the function of central and peripheral Avprlb renewed vigour. Avprlb KO mice together with the long-standing subject of Avp research, the Brattleboro rat, serve as robust systems with which to study the role of the Avprlb and Avp in the HPA axis response to stress.

## The HPA axis and stress

The complex homeostatic control that constantly acts to resist challenges and fluctuations in the internal environment that may threaten the survival of an organism is necessary for life. As a result of any deviation in conditions, a host of physiological and behavioural changes occur that allow an organism to adapt to such challenges and restore the homeostatic balance. One such neuroendocrine system that is activated in stressful circumstances is the HPA axis. The end product of HPA axis activation is an increase in circulating glucocorticoids that, together with other stress mediators, act on target cells to enable the organism to cope with the stress. Consequences of elevated glucocorticoids are widespread as cytosolic glucocorticoid receptors are present in most central and peripheral tissues. The most profound effects of elevated glucocorticoid levels are immunological and metabolic changes. Glucocorticoid secretion, in concert with catecholamine release due to rapidly activated sympatho-adrenomedullary system, prepares tissues for the physical “load” that may be required by the body as part of a coordinated response to manage or tackle the stress.

The HPA response to stress relies on several key mediators that fine-tune glucocorticoid release, which is dependent on a number of factors such as the nature of the stressor and the immunological state of the organism, tailoring the response to the specific stressor concerned. When subjected to a stressor, the challenge is perceived in brain regions appropriate to the nature of the stressor. Interpretation of these signals in relevant brain areas, such as various limbic or hindbrain regions, leads to activation (or inhibition) of the PVN (Herman et al. 2003).

The parvocellular subdivision of the PVN (pPVN) is the most important site among several hypothalamic nuclei that regulate the HPA response, as this is where stress signals are integrated and adjusted before peripheral signalling is initiated. Once activated, pPVN projections that terminate in the external zone of the median eminence release Avp and Crh into the hypophysial portal blood (Antoni 1993). These two ACTH secretagogues act on Avpr1bs and Crh type 1 receptors (Crhr1; coupled to G_s_ and adenylate cyclase activation) present in pituitary corticotrophs causing the release of ACTH into the peripheral blood system. ACTH via melanocortin receptors present in zona fasciculata cells of the adrenal cortex stimulates a rapid secretion of glucocorticoids into the peripheral blood supply. Glucocorticoids such as corticosterone ((CORT) in rodents) and cortisol (in humans) in turn provide negative feedback control of the HPA axis via pituitary, pPVN and higher brain centre glucocorticoid receptors (e.g. HIP).

The dominance of Crh as the primary ACTH secretagogue is still the prevailing view; however, numerous direct and indirect neuronal inputs into the pPVN, as well as humoral influences from blood or cerebrospinal fluid-borne stress signals, dynamically modulate the contribution of Crh to the stress response. The influence of Avp/Avpr1b may be of greater significance than that of Crh/Crhr1 in some stress circumstances, such as in response to some chronic (repeated) stressors (Ma et al. 1999) or to novel stressors superimposed on a repeated stress paradigm (Ma et al. 1997). Evidence of a switch from Crh-ergic to vasopressinergic pPVN drive in response to some chronic stressors reinforces the concept that each response is tailored to that specific stressor and that Avp may be an increasingly important mediator in these circumstances (Aguilera 1994; Ma et al. 1999; Aguilera et al. 2008). Moreover, studies of some specific acute stressors indicate that Avp may be preferentially released in favour of Crh (e.g. insulin-induced hypoglycaemia (IIH) in rats (Plotsky et al. 1985), ketamine anaesthesia and IIH in sheep (Engler et al. 1989)).

It is important to emphasise that in some species (e.g. sheep and horse) Avp rather than Crh appears to be the main ACTH secretagogue (Engler et al. 1989; Alexander et al. 1997).

## V1B receptor KO mice

With the generation of Avpr1b KO mice by us in 2002, attention was initially directed towards the deficits observed in cognitive and behavioural tests (Wersinger et al. 2002; Wersinger et al. 2004). It was noted that mice lacking functional Avpr1bs showed impairments in social recognition and reduced responses in some aggression paradigms whereas other physiological and behavioural test responses were normal (Wersinger et al. 2002, 2004, 2007a). Findings from a second Avpr1b KO line generated by Tanoue and co-workers initially focussed on the disruption of the HPA axis (Tanoue et al. 2004), and they reported that KO mice have lower circulating ACTH levels under basal and acute stress conditions. This is in contrast to basal measurements of HPA axis activity seen in our Avpr1b KO mouse colony, which maintain normal resting ACTH levels (Lolait et al. 2007a). Notwithstanding basal HPA axis differences, both Avpr1b KO lines have been used to generate a large amount of compatible evidence supporting the involvement of the Avpr1b in stress and aggression, which is also largely consistent with *in vivo* Avpr1b antagonism with SSR149415 (Griebel et al. 2002; Blanchard et al. 2005; Stemmelin et al. 2005).

### Reduced aggression in KO mice

Avp has been implicated as a moderator of several central behaviours that were initially thought, based on pharmacological profiles and due to its much higher prevalence in the brain, to be mediated by the Avpr1a. Experiments in rodents, particularly hamsters, with Avpr1a antagonists have consistently shown that Avpr1as facilitate some aggressive behaviour (Ferris et al. 1997, 2006; Caldwell and Albers 2004), although this is yet to be verified in Avpr1a KO mutants (Wersinger et al. 2007b). It is possible that the neural circuitry underlying aggression compensates for the loss of the Avpr1a in the global Avpr1a KO. In contrast, evidence of Avpr1b involvement in aggression comes from both pharmacological and KO data, e.g. antagonism of the Avpr1b with SSR149415 lowers the frequency and duration of aggressive behaviour in hamsters (Blanchard et al. 2005) while Avpr1b KO mice display reduced attack number and longer attack latency compared to wild types. This latter observation has been further categorised as a deficit in the attack component of aggression, as defensive aggression remains intact in mutant animals (Wersinger et al. 2002, 2007a). Furthermore, the reduced aggression phenotype persists when Avpr1b KO mice are crossed with a more aggressive wild-derived mouse strain, confirming that the reduced aggression observed is not simply a peculiarity of laboratory mouse strains (Caldwell and Young 2009). The specific neural substrate(s) vital for Avpr1b's role in aggression is not known, nor is the possible interaction between Avpr1a and Avpr1b (or Oxt and the Oxtr for that matter—see, Winslow et al. 2000) *in vivo.* It should be noted that the central distribution of Avpr1a- and Oxtr-binding sites and Avpr1b mRNA expression are clearly distinct but may overlap in some brain regions (e.g. olfactory system) (see, Table III in Beery et al. 2008; Caldwell et al. 2008a).

**Table III:** Summary of studies conducted with Avpr1b antagonists on HPA activity *in vivo*.

Index of HPA activity	Effect of antagonist	Compound/route/species	Reference
Basal ACTH/CORT	↔	Org/iv/rat	Spiga et al. (2009a)
Avp-induced ACTH release	↓	SSR*/ip * po/rat	Serradeil-Le Gal et al. (2002)
Avp-induced ACTH release	↓	SSR/iv/rat	Chen et al. (2008)
Avp-induced ACTH release	↓	Org/iv/rat	Spiga et al. (2009a)
Avp-induced ACTH release	↓	ABT-430 & ABT-558/po/mouse	Behl et al. (2008)
Avp-potentiated Crh-induced ACTH release	↓	SSR/po/rat	Serradeil-Le Gal et al. (2002)
d[Cha^4^]Avp†/Crh-induced ACTH release	↓	Org/po/rat	Craighead et al. (2008)
dDAVP‡/Crh-induced ACTH release	↓	Org/po/rat	Craighead et al. (2008)
Acute resgraint-induced ACTH release	↓	SSR/ip/rat	Serradeil-Le Gal et al. (2002)
Acute restraint-induced ACTH release	↓	SSR/sc/rat	Ramos et al. (2006)
Acute restraint-induced ACTH release	↓	Org/iv/rat	Spiga et al. (2009a)
Acute restraint-induced ACTH release	↓	ABT-430 & ABT-558/po/mouse	Behl et al. (2008)
Acute lipopolysaccharide (LPS)-induced ACTH release	↓	Org/iv/rat	Spiga et al. (2009a)
Acute ether-induced ACTH release	↓	SSR/ip/rat	Ramos et al. (2006)
Acute footshock-induced ACTH release	↓	SSR/ip/rat	Zhou et al. (2008)
Acute noise-induced ACTH release	↔	Org/iv/rat	Spiga et al. (2009a)
Acute forced swimming-induced ACTH release	↔	SSR/sc/rat	Ramos et al. (2006)
Chronic restraint-induced ACTH/CORT adaptation	↔	Org/sc/rat	Spiga et al. (2009b)
Sensitisation (↑) ACTH response to acute noise after acute or repeated restraint	↓	Org/sc/rat	Spiga et al. (2009b)
Sensitisation (↑;) ACTH response to acute hypertonic saline after repeated restraint	↔	SSR/po/rat	Chen et al. (2008)

Notes: ↓, Hormone values are reduced following Avpr1b antagonist administration; ↔, hormone values are unchanged following Avrp1b antagonist administration

* SSR = SSR149415

† d[Cha^4^]Avp = Avpr1b agonist

‡ dDAVP = Avpr2 agonist.

The changes in aggression, as well as differences in social motivation (Wersinger et al. 2004) or social memory evident in Avpr1b KO mice may be due to deficits in the processing of accessory olfactory stimuli that are needed to evoke such behaviours (Caldwell et al. 2008b). It is suggested that the role of central Avpr1bs may be to couple socially relevant cues detected in the accessory olfactory system to the appropriate behavioural response (Caldwell et al. 2008b). Intriguingly, both Avpr1a and Avpr1b genes are expressed in the forebrain olfactory system (Ostrowski et al. 1994; Hernando et al. 2001). Evidence of pyramidal CA2 Avpr1bs (Hernando et al. 2001; Young et al. 2006) also suggests a relationship between the deficits in social memory and the uncoupling of social cues from the accessory olfactory system and the formation or recall of relevant memories (Caldwell et al. 2008b). Based on studies in Avpr1b KO animals, the central Avpr1b may also be involved in a number of other behaviours (summarised in Table I). Prepulse inhibition of the startle reflex is attenuated in Avpr1b KO mice (Egashira et al. 2005) suggesting that this mouse may be a suitable model to investigate sensorimotor gating. In contrast, no major changes in anxiety-like or depression-like behaviours are observed in Avpr1b KO animals (Wersinger et al. 2002; Egashira et al. 2005; Caldwell et al. 2010). These results conspicuously differ from some of those obtained in Brattleboro rats (Mlynarik et al. 2007) or with Avpr1b antagonist administration, mainly in rats (see below), and strongly suggest that the Avpr1b KO mouse is not an appropriate model for examining stress-induced anxiety or depression (e.g. see Kalueff et al. 2007).

**Table I:** Behavioural studies conducted in Avpr1b KO and wild-type mice that suggest central participation of the Avpr1b.

Behaviour/process	Behavioural paradigm/test	Effect in Avpr1b KO	Reference
Aggression	Predatory (attack cricket)	↔	Wersinger et al. (2007a)
	Maternal (pup defence)	↓	Wersinger et al. (2007a)
	Competitive (food deprivation/competition)	↓	Wersinger et al. (2007a)
	Defensive (attack avoidance)	↔	Wersinger et al. (2007a)
	Defensive (retaliatory aggression when attacked)	↓	Wersinger et al. (2007a)
	Offensive (resident intruder)	↓	Wersinger et al. (2002)
	Offensive (neutral arena)	↓	Wersinger et al. (2002)
Memory/social memory & recognition	Social memory (Bruce effect)	↓	Wersinger et al. (2008)
	Social recognition (familiar female recognition)	↓	Wersinger et al. (2002)
	Morris water maze	↔	Wersinger et al. (2002)
	Social memory (littermate recognition vs. novel animal)	↓	DeVito et al. (2009)
	Temporal order memory	↓	DeVito et al. (2009)
Social motivation	Bedding preference	↓	Wersinger et al. (2004)
	Sociability (familiar littermate interaction)	↓	DeVito et al. (2009)
	Sociability (investigation of novel animal vs. novel object)	↔	Yang et al. (2007)
	Social dominance (mounting behaviour)	↓	Caldwell et al. (2010)
Anxiety-related behaviour	Ultrasonic vocalisation (nest separation/resident intruder)	↓	Scattoni et al. (2008)
	Anxiolytic effect of chronic SNRI treatment (open arm test)	↓	Ishizuka et al. (2010)
	Open-field, elevated plus maze	↔	Wersinger et al. (2002)
	Open-field, light/dark, elevated plus maze	↔	Egashira et al. (2005)
Depression-like behaviour	Forced swimming test	↔	Egashira et al. (2005)
		↔	Caldwell et al. (2010)
Influence of ethanol	Voluntary ethanol consumption	↔	Caldwell et al. (2006)
	Intoxicating effects of ethanol (e.g. motor co-ordination)	↔	Caldwell et al. (2006)
Prepulse inhibition	Prepulse inhibition of the acoustic startle reflex	↓	Egashira et al. (2005)
	Prepulse inhibition of the acoustic startle reflex + atypical antipsychotic drug administration	↔	Egashira et al. (2005)

Notes: Note that defensive aggression behaviours are less influenced by lack of Avpr1b than attacking behaviours. Furthermore, Avpr1b KO mice not only show clear deficits in social memory/recognition but also temporal order memory. Memory deficits are not attributable to a reduction in olfactory performance that suggests the detection of olfactory cues remains unaffected but an altered processing of olfactory cues results in the behaviour change. Interestingly, disruption in prepulse inhibition to acoustic startle seen in Avpr1b KO mice parallels that seen in some human conditions such as schizophrenia and panic disorders. Injection of Avpr1b KO mice with atypical antipsychotics used to treat schizophrenia appeared to reverse these deficits. Arrows indicate changes observed in the behavioural phenotype of Avpr1b KO mice compared to wild-type animals.

### The stress response in KO mice

The impact of Avp in ACTH release is often regarded as ancillary to Crh as Avp alone is a weak ACTH secretagogue but together with Crh it acts synergis-tically to facilitate ACTH secretion (Gillies et al. 1982; Rivier and Vale 1983; Antoni 1993). Not only do the corticotroph Avpr1b- and Crh-signalling pathways converge to increase ACTH release (Abou-Samra et al. 1986), the two receptors may physically heterodimerise (Young et al. 2007). We, and others, have subjected adult Avpr1b KO animals to a number of acute and chronic (repeated) stressors of varying severity and nature (Tanoue et al. 2004; Itoh et al. 2006; Lolait et al. 2007a,b; Stewart et al. 2008a,b). These studies clearly demonstrate that a functional Avpr1b is essential for mounting a normal HPA response, as manifested by increased plasma ACTH levels, to most acute stressors (summarised in Table II). The one exception is the response to “severe” restraint where there is no difference in either plasma ACTH level or CORT level between Avpr1b KO and wild-type mice (Lolait et al. 2007a). Studies in “severely” restrained Brattleboro rats reveal a similar picture (Zelena et al. 2004). It is likely that the restraint procedure employed was sufficiently stressful as to override any contribution from Avp (acting via the Avpr1b), e.g. ACTH secretion may be entirely dependent on Crh acting alone or in concert with other “minor” ACTH secretagogues such as angiotensin II, vasoactive intestinal peptide or serotonin (Carrasco and Van de Kar 2003). When comparing the reduced HPA axis response to other acute stressors in Avpr1b KO mice, we consistently find a decreased ACTH response but not always a corresponding lower CORT level. The nature of stressors to which both ACTH and CORT, ACTH but not CORT and neither ACTH or CORT responses are reduced in Avpr1b KO mice does not fall into any existing classification of stress. This work is in agreement with experiments in Brattleboro rats which suggest that the magnitude of Avp contribution is dependent on the context of the stressor (Zelena et al. 2009). As shown in Table II, we find some acute and chronic stressors are not influenced by the loss of the Avpr1b (e.g. acute severe restraint), some have a reduced ACTH response only (e.g. acute forced swimming stress) and some have both a reduced ACTH and CORT response in Avpr1b KOs compared to wild types (e.g. acute and repeated novel environment stress). Thus, how Avp influences the HPA axis response to stress could provide a further basis of stressor classification.

**Table II:** A summary of our recent studies with the Avpr1b KO colony demonstrating the effects of a number of stressors on plasma ACTH and CORT levels in wild type vs. Avpr1b KO mice.

Avpr1b KO vs. wild-type mice	Acute stress		Repeated stress*		
			
Stressor	ACTH	CORT	ACTH	CORT	Adaptation in wild type
Severe restraint† (Lolait et al. 2007a)	↔ (♂)	↔ (♂)	↓ (♂)	↔ (♂)	No
Mild restraint‡ (Stewart et al. 2008a)	↓ (♂)	↔ (♂)	↓ (♂)	↔ (♂)	No
Forced swimming (Stewart et al. 2008a)	↓ (♂)	↔ (♂)	↓ (♂)	↔ (♂)	No
Novel environment (Stewart et al. 2008a)	↓ (♂)	↓ (♂)	↓ (♂)	↓ (♂)	No
Shaker stress (see Figure 2)	↓ (♂)	↔ (♂)	↔ (♂)	↔ (♂)	Yes (ACTH)
Lipopolysaccharide (LPS) (Lolait et al. 2007b)	↓ 30min and 4h (♀)	↓ 30 min, ↔ 4h(♀)			
Ethanol (Lolait et al. 2007b)	↓ (♂+♀)	↓ (♂+♀)			
Insulin-induced hypoglycaemia (Lolait et al. 2007a)	↓ (♂)	↓ (♂)			
Antidepressants (Stewart et al. 2008b)	↓ (♂+♀)	↓ (♂+♀)			

Notes: This table highlights how the nature of the stressor can influence the effects of the Avpr1b on ACTH and CORT release. Stressors that give rise to mismatches between ACTH and CORT data, and stressors that have shown some level of adaptation after chronic administration are shown. ↓, Hormone values are reduced in KO mice compared to wild-type mice; ↔, hormone values are unchanged in KO mice compared to wild-type mice

* Plasma hormone levels measured following the final acute stress in a 10–14 days daily repeated acute stress paradigm

† “Severe” restraint, mice placed in a 50 ml falcon tube with tissue paper packing inserted to restrict any movement of the animal

‡ “Mild” restraint, mice placed in 50 ml falcon tube.

Our studies in Avpr1b KO mice (Lolait et al. 2007a,b; Stewart et al. 2008a,b) and similar studies on acute stress in Brattleboro rats (Domokos et al. 2008; Zelena et al. 2009) both often note a disparity between stress-induced ACTH versus CORT release in Avp/Avpr1b-deficient animals. It is important to recognise that the CORT response to ACTH saturates at low circulating ACTH levels (Dallman et al. 2002). In addition, plasma hormones levels were only measured at one time point in our studies so any incremental change in CORT levels may have been missed. Furthermore, we have assumed that the dynamics of stress-induced Avp, Crh and/or other ACTH secretagogue is similar in Avpr1b and wild-type mice. Where stress-induced ACTH release in Avpr1b KO mice is not always followed by a proportional CORT attenuation, suggests that CORT may be released independently of ACTH in some circumstances. Interestingly, this pattern of ACTH/CORT profiles in response to acute stress also appears to be present in neonatal Brattleboro rats (Zelena et al. 2008). Discrepancies between plasma stress hormone levels appear stressor specific suggesting that a particular stressor may possess specific characteristics that could activate a number of pathways to promote CORT release, e.g. via direct splanchnic or other neural innervation of the adrenal cortex or medulla (Ehrhart-Bornstein et al. 1998). Alternatively, adrenal sensitivity to locally synthesised or humoral factors may be altered permitting CORT release even when low levels of circulating ACTH are present. Adrenal hypersensitivity and ACTH-independent pathways of CORT release would likely bypass the normal feedback controls that govern ACTH release from the pituitary (for review see Bornstein et al. 2008).

As mentioned above, chronic (repeated) stress has often been associated with an alteration in the control of ACTH release from what is a predominantly Crh-mediated drive, seen in acute stress, to an Avp-mediated drive speculated to maintain ACTH levels during adaptation to repeated stress (Harbuz and Lightman 1992; Aguilera 1994; Ma et al. 1997; Aguilera and Rabadan-Diehl 2000). Adaptation to repeated stress (i.e. lower ACTH and/or CORT following repeated stress compared to a single episode of acute stress) is species and stressor specific and is not always observed (Marti and Armario 1998; Armario et al. 2004). The apparent flip in control of ACTH release from Crh to Avp that is seen in some repeated stressors in rats (e.g. restraint) has made Avp and the Avpr1b attractive targets for pharmacological intervention in conditions of repeated or chronic stress, although it should be noted that Avp does not play a role in HPA axis responses to all chronic stressors (e.g. chronic morphine injection (Domokos et al. 2008)). The preferential expression of Avp in the pPVN in adaptation to chronic stress observed in rats is associated with an upregulation of Avpr1bs (but not Crh receptors) in the anterior pituitary gland (Aguilera 1994). Chronic stress from repeated (daily) exposure to an acute stressor leading to ACTH hyperresponsiveness to a single, novel stressor episode may also involve an increase in PVN Avp (Ma et al. 1999) and pituitary Avpr1b expression, and pituitary ACTH hyperresponsiveness (Aguilera 1994). However, Avp/Avpr1b does not appear to be responsible for HPA axis hypersensitivity to novel stressors (Chen et al. 2008). The mechanism(s) by which hypothalamic Avp and pituitary Avpr1b responsiveness is maintained during stress adaptation suggests the existence of numerous transcriptional and translational regulatory components involved in PVN and pituitary plasticity that dynamically alter Avp and Avpr1b levels according to demand (Volpi et al. 2004a,b). One view is that Avp/Avpr1b may alter corticotroph proliferation and pituitary remodelling during prolonged activation of the HPA axis (Subburaju and Aguilera 2007). However, studies of repeated stress in Avpr1b KO mice and Brattleboro rats, suggest that the role of the Avpr1b and its cognate ligand in the adaptation of ACTH/CORT levels to chronic stress may not be as convincing as first thought.

**Figure 2:**
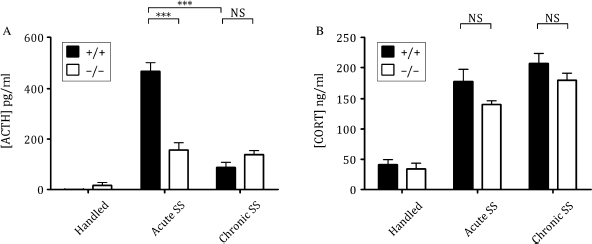
Plasma hormone levels of male wild type (+/+) and KO (2 /2) mice following acute and chronic (repeated) SS. Note that the ACTH response to acute SS in wild type but not Avpr1b KO animals is significantly reduced following repeated SS. Mice of appropriate genotype generated from parental crosses heterozygous for the Avpr1b were randomly assigned to an experimental group containing 7–8 mice. SS consisted of shaking mice in separate clean empty cages on a standard bench top orbital shaker (stroke: 38 mm at 150 rpm) for 10 min. Mice were returned to their home cage for a further 10 min prior to sacrifice. Chronically shaken mice were stressed once daily for 10 days whereas mice in acute groups were handled for 9 days and stressed once on the 1 0th day. Control mice were handled once daily and all experiments were performed between 0900 and 1130h in accordance with UK Home Office regulations (Animal Scientific Procedures Act (1986)) and approved by the University of Bristol Ethical Review Board. Plasma ACTH (graph A) and CORT levels (graph B) were determined by ELISA and EIA, respectively (IDS, Tyne and Wear, UK). Data are expressed as mean ± SEM. Significant differences are denoted as ***: *P* < 0.001, NS: not significant.

For the chronic (repeated) stressors tested in Avpr1b KO mice, there is a reduction in the ACTH response to a final acute stress following 10–14 days of stress repeated once daily. The responses of Avpr1b KO animals and wild-type animals exposed to repeated stress are summarised in Table II. These studies have a number of salient features: firstly, as observed in male Brattleboro rats subjected to repeated restraint (Zelena et al. 2004), the reduction in the ACTH response following repeated stress in Avpr1b KO animals is not often accompanied by a similar reduction in CORT responses—this mirrors what we have observed in these animals' responses to acute stress (see above). Secondly, with the exception of the ACTH response to repeated, severe restraint, the ACTH and CORT responses to acute or repeated stress are of equivalent magnitude. This suggests that the Avpr1b participates in the fast ACTH secretion (as seen in the response to acute stress) in repeatedly stressed mice. And finally, of all the repeated stressors studied in wild-type mice from our Apr1b KO colony, adaptation in ACTH responses was only seen with repeated exposure to shaker stress (SS) (Figure 2). No adaptation in CORT responses was observed in Avpr1b or wild-type mice following any repeated stress paradigm. There is a robust plasma ACTH and CORT increase in response to a single, acute SS episode in wild-type mice; however, after 10 days of repeated SS, the ACTH response is reduced in these animals (see, Figure 2—graph A: plasma ACTH levels, wild-type acute stress response vs. wild-type chronic stress response). The acute ACTH response in Avpr1b KO mice, while reduced from that seen in wild-type animals, is the same for repeated SS. Furthermore, the ACTH response to repeated SS in Avpr1b KO mice is not different from that seen in wild-type mice. It is tempting to speculate that there is a loss of adaptation to repeated SS in Avpr1b KOs but such a conclusion would be tenuous since ACTH levels in acutely stressed Avpr1b KO mice are already very low. The lack of functional Avpr1b in the KO mice has such a profound effect on the ACTH response to SS in these mice that any non-Avpr1b-mediated adaptation to repeated SS is probably negligible. The studies in Avpr1b KO mice and Brattleboro rats highlight the discrepancy between Avp/Avpr1b-mediated ACTH and CORT secretion during acute and repeated stress—this may have implications on the potential use of Avpr1b antagonists to ameliorate symptoms of HPA axis hyperactivity in stress-related disorders.

### Gene expression in Avpr1b KO mice

**Figure 3:**
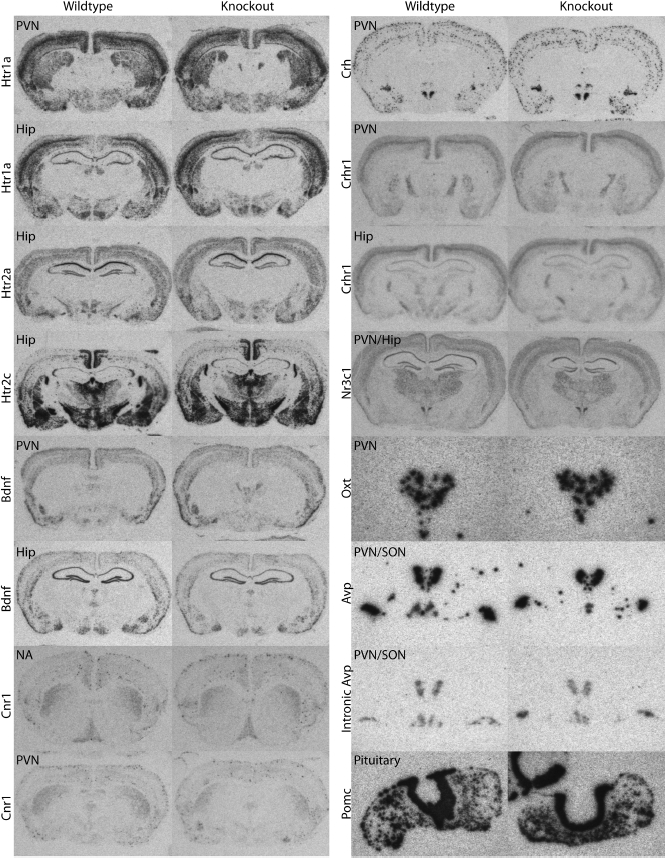
Representative ISHH photomicrographs of gene expression in brain and pituitary of male Avpr1b KO and wild-type mice. Riboprobes corresponding to the following sequences were used: 5-HT1a receptor (Htr1a) (bp1230–1597 of Genbank Accession number NM_008308), 5-HT2a receptor (Htr2a) (bp1884–2492 of Acc#NM_172812), 5-HT2c receptor (Htr2c) (bp1435–1965 of Acc#NM_008312), brain-derived neurotrophic factor (Bdnf) (bp310–822 of Acc#AK017559), cannabinoid CB1R (Cnr1) (bp728–1040 of Acc#Y18374), Crh (bp101–686 of Acc#205769), type 1 Crh receptor (Crhr1) (bp160–528 of Acc#NM_007762), glucocorticoid receptor (Nr3c1) (bp439–958 of Acc#X04435), Oxt (bp1753–1865 of Acc# M88355), Avp (bp2966–3388 of Acc# M88354), intronic Avp (bp1965–2406 of Acc#M88354) and pro-opiomelanocortin (Pomc) (bp145-620 of Acc#V01529). The intronic Avp probe was targeted towards intron 1 of the Avp/neurophysin II gene to reflect heteronuclear RNA expression levels. These probes were obtained by PCR using genomic DNA, or brain (Crhr1, Crh, Bdnf, Avp and Oxt) or pituitary (Pomc) cDNA from 129 Sv mouse tissue as template. Restriction sites were incorporated into the 5′ ends of PCRprimers to facilitate cloning of the PCRproduct into vector pGEM4Z. The integrity of all riboprobes was verified by DNA sequencing. ISHH on 12 μm sections with ^35^S-labelled riboprobes was performed as in ref. (Lolait et al. 2007a). Sections were exposed to X-ray film (Hyperfilm MP, Amersham; GE Healthcare, Little Chalfont, UK) for hours (Pomc, Avp & Oxt) to weeks. Sections hybridised with the corresponding sense probes gave negligible, background staining (not shown). Brain regions shown include the nucleus accumbens (NA), PVN, supraoptic nucleus (SON) and the HIP. Sections of brain hybridised with the Nr3c1 probe were cut at a greater pitch around a mediolateral axis to obtain both HIP and PVN in one slice. Photomicrographs were taken at 6.3 × magnification and resized for publication (except Oxt, Avp and intronic Avp probes which are magnified appropriately to show the PVN or PVN/SON in greater detail).

Whether the deficits observed in Avpr1b KO mice outlined above are a direct result of disruption of Avpr1b signalling pathways or the result of an altered compensatory expression profile is not known. As far as HPA axis function is concerned, clearly Crh (or Oxt) does not compensate for the loss of the Avpr1b in Avpr1b KO mice. There are some direct changes that occur in KO mice that give rise to phenotypes such as altered glucose metabolism and attenuated ACTH release; however, indirect changes that affect behavioural systems that may lead to the deficits seen in Avpr1b KOs have yet to be identified. We have used ISHH to assess basal gene transcript levels of a number of genes that are closely linked with HPA axis function and find no significant differences in gene expression between Avpr1b KO and wild-type mice (Figure 3). Comparisons of Oxtr mRNA levels in Avpr1b KO and wild-type mouse anterior pituitaries appear to suggest an upregulation of Oxtrs in Avpr1b KO mice (Nakamura et al. 2008). As Oxt at high concentrations can elicit ACTH release via the Avpr1b, and the Oxtr may be expressed in corticotrophs, it has been suggested that increased expression of Oxtrs may be a compensatory mechanism through which Avpr1b KO mice and Brattleboro rats can, to some degree, make up for the lack of Avpr1b/Avp-mediated ACTH release (Nakamura et al. 2008). We cannot totally rule out the possibility that mechanisms compensating for the loss of Avpr1b are active in the Avpr1b KO. Changes in neurochemical networks active centrally (e.g. projections to the PVN or signals within the PVN itself) or at the level of the anterior pituitary and adrenal may have occurred. We also cannot exclude a role of central Avpr1bs (or for that matter Avpr1as) in directly or indirectly influencing HPA axis activity. Nevertheless, stress-induced ACTH levels in our Avpr1b KOs are consistently lower (often to basal levels) than wild-type controls, confirming any compensation (e.g. from the action of Crh) is not sufficient to fully counteract the loss of the Avpr1b (Lolait et al. 2007a,b; Stewart et al. 2008a,b). The role of potential Oxtr-mediated ACTH release in Avpr1b KO mice and Brattleboro rats remains to be clarified, however, as the Oxtr is predominantly located in lactotrophs rather than corticotrophs in the anterior pituitary (Breton et al. 1995) it is likely that the Oxtr plays a minor role compared to the Avpr1b in wild-type animals. The use of conditional KOs and emerging pharmacological developments will no doubt help clarify the contribution of the pituitary Oxtr in acute and chronic stress.

### Emerging pharmacological data

A number of agonists (such as desmopressin, an Avpr2 agonist) and antagonists (such as relcovaptan, an Avpr1a antagonist) are available and frequently used to study the Avpr1a, Avpr2 and Oxtr (for review see Lemmens-Gruber and Kamyar 2006; Manning et al. 2008). Some of these are in clinical trials or have been approved for use as treatments in disorders such as nocturnal enuresis and neurogenic diabetes insipidus. The search for Avpr1b pharmacological tools has gained much impetus due to their potential as treatments for conditions associated with chronic stress such as anxiety and depression (for short review see Griebel et al. 2005; Arban 2007). The most widely used non-peptide Avpr1b antagonist, SSR149415, has been used extensively in research since its characterisation in 2002 (Serradeil-Le Gal et al. 2002) (see, Tables III and IV). SSR149415 is orally active and inhibits some but not all acute stressor-induced ACTH release in rats (Serradeil-Le Gal et al. 2002; Ramos et al. 2006), and does not affect HPA hypersensitivity to novel stressors (Chen et al. 2008). The compound acts at the human Avpr1b and also has some antagonist activity at the human Oxtr *in vitro* (Griffante et al. 2005) but has high selectivity and nanomolar affinity for rodent forms of the Avpr1b (Serradeil-Le Gal et al. 2002). In mice and rats, SSR149415 has been tested in a variety of classical models of anxiety (e.g. elevated plus maze, light/dark box test) and depression (e.g. forced swim test, chronic mild stress) as well as other models (e.g. olfactory bulbectomy, Flinder's sensitive line) that are used to determine the efficacy of potential anti-depressant and anxiolytic drugs (Griebel et al. 2002; Overstreet and Griebel 2005; Stemmelin et al. 2005; Louis et al. 2006; Salomé et al. 2006; Shimazaki et al. 2006; Hodgson et al. 2007; Iijima and Chaki 2007). Peripheral and central pretreatment with SSR149415 reduces anxiety and depressive-related behaviour in these tests with high compatibility between the findings of these studies. SSR149415 also reduces aggression in hamsters (Blanchard et al. 2005), significantly reverses the reduction in dentate gyrus cell proliferation caused by chronic mild stress in mice (Alonso et al. 2004) and blocks stress-induced hyperalgesia in rats (Bradesi et al. 2009). It has also been radiolabelled with tritium and used in receptor autoradiography to reveal low-resolution binding in the human and rat pituitary—no Avpr1b binding sites

were observed in sections of rat brain (Serradeil-Le Gal et al. 2007). Recently, SSR149415 has failed phase II clinical trials (Kirchhoff et al. 2009). Overall, the results of studies with SSR149415 evidence a possible role for the Avpr1b in affective disorders and point to animal model-validated targets with which to treat them.

Around the same time, SSR149415 was first reported as an Avpr1b antagonist, the Avp peptido mimetic [1-deamino-4-cyclohexylalanine] arginine vasopressin (d[Cha^4^]Avp) was described (Derick et al. 2002). This agonist was the first to show efficacy at nanomolar concentrations and stimulate the release of ACTH/CORT without exhibiting vascular or renal activity (Derick et al. 2002). Other peptide agonists selective for the rat Avpr1b have been generated by modifying positions 4 and 8 of the Avp analogue deamino-[Cys] arginine vasopressin (Pena et al. 2007a). Many of these modified Avp analogues display high selectivity for the Avpr1b and bind with sub-nanomolar affinities (Pena et al. 2007a) and thus could well be useful in the study of the Avp receptors in rodents; however, they may be of limited use as human therapeutics due to their peptidergic nature. The agonists created since d[Cha^4^]Avp do, however, have an increasingly refined agonist profile. One such member of the recent modified range of Avp analogue agonists, d[Leu^4^,Lys^8^] Avp, is noted to be a full agonist at human, rat and mouse Avpr1bs *in vitro* as well as stimulates ACTH and insulin release at low doses from mouse pituitary and perfused rat pancreas, respectively (Pena et al. 2007b). This effect of d[Leu^4^,Lys^8^]Avp on mouse and rat tissue is blocked when co-administered with SSR149415 (Pena et al. 2007b).

Since the development of SSR149415, there have been some recent reports of a non-peptide antagonist (Org) that is highly selective for the human and rat Avpr1b (Craighead et al. 2008). Pretreatment of rats with this compound causes a significant reduction in ACTH release after restraint stress or lipopolysaccharide (LPS) challenge (Spiga et al. 2009a) and to a heterotypic stressor after repeated restraint (Spiga et al. 2009b) (see Table III). However, Org does not affect repeated restraint stress-induced ACTH/-CORTadaptation in rats (Spiga et al. 2009a). Another set of Avpr1b antagonists (ABT-436 and ABT-558) have subnanomolar affinity for the human Avpr1b with approximately 30-fold lower affinity for rat and mouse Avpr1bs (Wernet et al. 2008). These compounds attenuate acute restraint stress-induced ACTH release (Behl et al. 2008) and appear to have increased anxiolytic-like and antidepressant-like potency and efficacy compared to SSR149415 (van Gaalen et al. 2008). More recently, other non-peptide antagonists have been described: “p”, a tetrahydroquinoline sulphonamide derivative, with high selectivity for the rat and human Avpr1b (K_i_s approximately 21 nM and 44 nM, respectively) (Scott et al. 2009), and compounds generated from a series of pyrrole-pyrazinone and pyrazole-pyrazinone derivatives which also appear to show good selectivity and high potency (e.g., compound 11 pIC_50_ = 8.4) for the human Avpr1b expressed in Chinese hamster ovary cells (Arban et al. 2010)—to our knowledge the effects of these compounds on HPA axis activity or behaviour have not been reported to date.

In conclusion, the distribution and functional studies of the Avpr1b have established its major role in the pituitary where it plays a pivotal part in the regulation of the HPA response to stress, and in particular to acute stress. Additionally, we see several, perhaps minor, metabolic and endocrine roles in the periphery. Behavioural changes generated from experiments in Avpr1b KO animals, together with recent Avpr1b antagonist data, have highlighted a more elusive central role for this receptor. The behavioural implications of Avp, acting via the Avpr1b, in aggression and stress, and the integral connection between stress, anxiety and depression make the Avpr1b an attractive target for pharmacological intervention. Increased Avp secretion and enhanced pituitary responsiveness to Avp have been reported in some subtypes of depression (e.g. melancholic depression)(see, Dinan and Scott 2005 for a review). Furthermore, polymorphisms in the Avpr1b gene have been associated with depression (van West et al. 2004), childhood-onset mood disorder (Dempster et al. 2007) and attention-deficit hyperactivity disorder (van West et al. 2009). The progress made in generating compounds selective for this receptor may have considerable implications for potential treatments for a number of disease states as well as for Avp research in general. The development of new compounds that can be radiolabelled to high specific activity is a critical step in Avpr1b research as determination of its central distribution may well provide an anatomical template to assign how changes in behaviours or disease states are influenced. Further developments in molecular (e.g. conditional Avpr1b KOs; the use of Avpr1b-specific small-interfering RNAs to selectively silence Avpr1b gene expression in specific brain regions) and pharmacological tools for use in rodents and primates (e.g. positron emission topography (PET) ligands, see Schonberger et al. 2010) will help elucidate the full function of the Avpr1b and thus the therapeutic value research into this receptor may hold.

## Figures and Tables

**Table IV:** Summary of studies conducted with Avprlb antagonists on behaviour.

Behaviour/process	Behavioural paradigm/test	Effect of antagonist	Compound/route/species	Reference
Aggression	Offensive (resident-intruder)	↓	SSR*/po/hamster	Blanchard et al. (2005)
Anxiety-related behaviour	Four-plate test	↓	SSR/po/mouse	Serradeil-Le Gal et al. (2002)
	Punished drinking conflict test	↓	SSR/ip/rat	Griebel et al. (2002)
	Punished drinking conflict test	↔	SSR/intraseptal/rat	Stemmelin et al. (2005)
	Elevated plus maze	↓	SSR/po/rat	Griebel et al. (2002)
	Elevated plus maze	↔	SSR/intraseptal/rat	Stemmelin et al. (2005)
	Elevated plus maze	↓	SSR/basolateral amygdala/rat	Salomé et al. (2006)
	Elevated plus maze	↓	SSR/ip/rat	Hodgson et al. (2007)
	Elevated plus maze	↓	SSR/dorsal (but not ventral) HIP/rat	Engin and Treit (2008)
	Light/dark test	↓	SSR/ip/mouse	Griebel et al. (2002)
	Social defeat	↓	SSR/po/mouse	Griebel et al. (2002)
	Defence test battery	↓	SSR/po/mouse	Griebel et al. (2002)
	Social interaction test	↓	SSR/ip/rat	Overstreet and Griebel (2005)
	Social interaction test	↓	SSR/po/rat	Shimazaki et al. (2006)
	Separation-induced pup vocalisations	↓	SSR/ip/rat	Hodgson et al. (2007)
	Conditioned lick suppression	↓	SSR/ip/rat	Hodgson et al. (2007)
	Marble burying	↔	SSR/ip/mouse	Hodgson et al. (2007)
	Vogel conflict test	↔	SSR/ip/rat	van Gaalen et al. (2008)
	Vogel conflict test	↓	ABT-436 & ABT-558/ip/rat	van Gaalen et al. (2008)
Depression-like behaviour	Forced swim test	↓	SSR/po/rat	Griebel et al. (2002)
	Forced swim test	↓	SSR/central nucleus of amygdala/rat	Salomé et al. (2006)
	Forced swim test	↓	SSR/intraseptal/rat	Stemmelin et al. (2005)
	Forced swim test	↔	SSR/ip/mice & rat	Hodgson et al. (2007)
	Forced swim test	↓	SSR/ip/rat	van Gaalen et al. (2008)
	Forced swim test	↓	ABT-436 & ABT-558/ip/rat	van Gaalen et al. (2008)
	Forced swim test (Flinder's sensitive line)	↓	SSR/ip/rat	Overstreet and Griebel (2005)
	Chronic mild stress	↓	SSR/ip/mouse	Griebel et al. (2002)
	Chronic mild stress	↓	SSR/ip/mouse	Alonso et al. (2004)
	Olfactory bulbectomy-induced hyperactivity	↓	SSR/po/rat	Iijima and Chaki (2007)
	Olfactory bulbectomy-induced hyperactivity	↓	SSR/ip/rat	van Gaalen et al. (2008)
	Olfactory bulbectomy-induced hyperactivity	↓	ABT-436/ip/rat	van Gaalen et al. (2008)
	Tail suspension test	↔	SSR/ip/mouse	Hodgson et al. (2007)
	Differential reinforcement of low-rate 72 s (DRL-72 s)	↓	SSR/ip/rat	Louis et al. (2006)
Other	FG 7142 (anxiogenic benzodiazepine inverse agonist)—induced central Ach	↓ (no effect of central norepinephrine)	SSR/ip/rat	Claustre et al. (2006)
	Heroin seeking during tests of reinstatement induced by footshock or heroin primes	↓	SSR/ip/rat	Zhou et al. (2008)
	Stress-induced hypothermia	↔	SSR/ip/rat	van Gaalen et al. (2008)
	Stress-induced hypothermia	↓	ABT-436 & ABT-558/ip/rat	van Gaalen et al. (2008)
	Stress-induced visceral hyperalgesia	↓	SSR/ip/rat	Bradesi et al. (2009)

Notes: ↓, Decreased behavioural phenotype in animals; ↔, no behavioural change

* SSR = SSR149415.

## Notice of correction

Please note that the following amendments have been made to this paper, first published Online Early 9 September 2010: the removal of a duplication of text within Table I caption on page 6; in Table IV on page 12, the heading ‘Anxiety related behaviour’ has been moved up to the 2nd line in column one from its previous position at line 12; and a spelling error, incorrect year of publication and incorrect volume details have been amended in the Zhou et al. 2008 reference list and citations in Tables III and IV.
